# Haematological Profile and Intensity of Urogenital Schistosomiasis in Ghanaian Children

**DOI:** 10.1155/2017/4248325

**Published:** 2017-06-21

**Authors:** Justice Afrifa, Desmond Gyedu, Eric Ofori Gyamerah, Samuel Essien-Baidoo, Isaac Mensah-Essilfie

**Affiliations:** ^1^Department of Medical Laboratory Technology, University of Cape Coast, Cape Coast, Ghana; ^2^Department of Biochemistry, University of Cape Coast, Cape Coast, Ghana; ^3^Department of Mathematics and Science Education, University of Cape Coast, Cape Coast, Ghana

## Abstract

**Background:**

Urogenital schistosomiasis is a widely contracted parasitic helminth infection often associated with haematological abnormalities.

**Aim:**

We investigated the relationship between the haematological profile and the intensity of schistosomiasis among children in the Yeji district.

**Materials and Methods:**

A total of 100 participants comprising 50* Schistosoma haematobium* (*S. haematobium*) infected and 50 noninfected controls aged 6–17 years matched for age and sex were recruited into the study. Blood and urine samples were collected and haematological profile and presence of* S. haematobium* eggs were assessed using standard protocols.

**Results:**

Haemoglobin (HGB) (*P* < 0.0001), haematocrit (HCT) (*P* < 0.0001), mean cell volume (MCV) (*P* = 0.0053), mean cell haemoglobin (MCH) (*P* < 0.0001), and mean cell haemoglobin concentration (MCHC) (*P* = 0.005) levels were reduced in cases compared to controls. Mixed cell percentage (MXD) (*P* = 0.018) and red blood cell distribution width (RDW-CV) (*P* = 0.012) were significantly elevated among cases as compared to controls. Haematuria was a clinical characteristic of heavy infection.

**Conclusion:**

* S. haematobium* infection creates an imbalance in the haematological profile. We found low HGB, HCT, MCV, MCH, and MCHC levels coupled with increased % MXD count and RDW-CV. Also, low MCV, MCH, and MCHC and high % MXD count are independently associated with* S. haematobium* infection among our study participants.

## 1. Introduction

High prevalence and intensity of* S. haematobium* infection usually occur in school age children resulting in increased morbidity and mortality among affected populations. Majority of* S. haematobium *infections are asymptomatic; therefore persons living in low resource areas only visit healthcare facilities after complications have developed [1]. Clinical presentations such as dysuria, haematuria, pyuria, and lesions of the bladder have been implicated in urinary schistosomiasis [2]. Iron deficiency anaemia is one major haematological presentation of heavy* S. haematobium* infection [3]. In acute cases, eosinophilia and leukocytosis have mostly been reported. In hepatosplenic patients, anaemia is common, usually normochromic and often macrocytic, likewise a significant rise in monocytes, lymphocytes, and neutrophils [4]. Meanwhile, a previous study [5] has also reported mild eosinophilia, leukocytosis, and mild neutropenia rather than anaemia as a feature of acute urogenital schistosomiasis. Haemoglobin, white blood cell (total) counts, neutrophil, and lymphocyte levels have also been reported to be normal among primary school children in Ibadan (Nigeria) [6]. Infection with* S*.* haematobium *influences the normal levels of certain blood constituents and treatment with praziquantel has been effective in normalizing the physiological conditions [1, 4, 7]. Interestingly, a significant relationship between the degree of eosinophilia and the intensity of infection has also been reported [4, 7].

In Ghana, earlier studies [4, 8, 9] have reported on the prevalence of* S. haematobium* infection. However, limited data exist on the effect of this infection on haematological indices. Also, most of these were cross-sectional studies which attempted to address the interactions between schistosomiasis and anaemia but have rather generated some conflicting results which render the magnitude of the relationship unclear. This study therefore examined the relationship between the haematological profiles and the intensity of urogenital schistosomiasis among primary school children in Yeji, Brong Ahafo Region, Ghana, using a case-control study design.

## 2. Material and Methods

### 2.1. Study Design/Settings

This community based case-control study was conducted from January to March 2015 at St. Mathias Catholic Hospital, Yeji, in the Atebubu District of the Brong Ahafo Region. Yeji is an island fishing port, lying on the southern bank of the White Volta in the Brong Ahafo Region. Brong Ahafo has an estimated population of 2,282,128 with Yeji being 129,248 and covers a total land mass of 39,557 square kilometers (population census of Ghana, 2010). The primary source of water for most of the inhabitants is mainly from the Volta Lake.

### 2.2. Study Population

A total of 179 school children aged 6–17 years were randomly recruited for this study out of which 140 met the criteria for inclusion. Of this number, 20 refused to participate, 12 did not give informed written consent, and another 7 refused to give blood samples for the study. The remaining participants included 50 children diagnosed with urogenital schistosomiasis (through standard light microscopy as cases), and 50 apparently healthy children (who tested negative for urogenital schistosomiasis) as controls. A standard pretested questionnaire was used to obtain demographic data as well as water contact activity.

### 2.3. Ethical Considerations

Ethical approval was obtained from the Institutional Review Board of the University of Cape Coast (UCCIRB) and the institutional ethics committee of the hospital. Written informed consent was obtained from guardians or teachers of eligible participants.

### 2.4. Inclusion and Exclusion Criteria

Healthy children matched for age and sex who visited the hospital for regular medical check-up were recruited as controls. Children who were on antihelminths drugs and those with conditions such as anaemia, intestinal parasitic infection, and other haemoglobinopathies such as sickle cell anaemia, thalassaemia, and leukemia were excluded from the study.

### 2.5. Blood Sample Collection

About three milliliters (3 ml) of venous blood was collected from participants into dipotassium ethylenediaminetetra acetic acid (K_2_ EDTA) tube for haematological analysis.

### 2.6. Haematological Analysis

Haematological profile (HGB, HCT, red cell indices, white blood cell count, platelet and white blood cell differential count) was estimated using an automated haematology analyzer (Sysmex KX-21N, Japan).

### 2.7. Urine Collection and Processing

Participants provided 10–20 ml freshly voided urine in clean, wide mouth and leak proof containers. Urine was screened for* S. haematobium *using centrifugation technique as described elsewhere [10].

### 2.8. Parasitological Examination

The urine samples were examined for the eggs of* S. haematobium *with a 10x objective. The number of* S. haematobium* eggs per 10 ml urine was determined for each participant as described earlier [11]. The number of eggs per slide was counted, and the infection intensity was categorized as light (<50 eggs/10 ml of urine) or heavy (≥50 eggs/10 ml of urine) as defined by the World Health Organization [12].

### 2.9. Statistical Analysis

Independent sample *t*-test was used to compare mean scores between* S. haematobium* infected subjects and controls. Chi-square was used to test the association between proportions of categorical variables. Correlations between* S. haematobium* infection and haematological profile were performed using Pearson's correlation coefficient test. *P* < 0.05 were considered statistically significant. Data was analyzed with SPSS version 16 (SPSS Inc., Chicago).

## 3. Results

A total of one hundred (100) participants of ages 6–17 were recruited into the study. Of the 100, 50* S. haematobium* infected children (cases) and 50 apparently healthy controls participated in the study. The cases included 27 females and 23 males. The mean age of the cases and the controls were 10.42 ± 2.16 years and 11.18 ± 3.20 years, respectively. We observed that the mean HGB, HCT, MCH, MCHC, MXD%, and RDW-CV were lower in the cases compared to the controls (*P* < 0.05). However, the WBC count and PLT were slightly but insignificantly elevated in the cases compared to controls (*P* > 0.05). Noticeably also, RBC, MCV, LYM%, and NEUT% were reduced in cases compared to the controls (Table 1).

Haematuria was significantly associated with heavy infection (*P* < 0.0001) (Table 2).

Multivariate logistic regressions of factors associated with* S. haematobium* infections as shown in Table 3 reveal that MCV < 85 [OR = 5.75 (95% CI = 1.19–27.81, *P* = 0.030)], MCH < 27 [OR = 8.81 (95% CI = 2.40–32.41, *P* = 0.001)], and MXD > 13 [OR = 2.50 (95% CI 1.02–6.15, *P* = 0.046)].

## 4. Discussion

Urogenital schistosomiasis caused by* Schistosoma haematobium* is one of the major neglected tropical diseases causing public health and socioeconomic problems and affecting about 700 million people worldwide [13]. We assessed the association between the haematological profile and the intensity of* S. haematobium* infection among school age children in the Yeji municipality, Ghana. Our results showed that children with* S. haematobium* infections had low HGB, HCT, MCV, MCH, and MCHC. Total WBC and WBC differential count (MXD and RDW-CV) were higher among* S. haematobium* infected children. We also report that low MCV, MCH, and high % MXD counts are independently associated with* S. haematobium* infections.

The association between* S. haematobium *and HGB concentration has been studied extensively [3, 4] and low levels of HGB have been suggestive of anaemia among people in endemic areas. In our study, participants with* S. haematobium* infection were more anaemic than the healthy controls (Table 1) which is in agreement with earlier findings [1, 4].

Interestingly, HGB levels were reported to be within normal ranges among school aged children infected with* S. haematobium* in Ibadan City (Nigeria) [6]. There have also been reports of* S. haematobium* infected children with low HGB who did not recover until six weeks after treatment in Kenya [14]. In this study, HGB concentration for the cases was below the normal reference limit as compared to the control group. Anaemia due to diminished production and increased loss of RBC could also account for the reduced HGB [15].

We also report RBC indices (MCV, MCH, and MCHC) below normal range in* S. haematobium* infected children compared to control. This agrees with previous studies [16, 17] which also reported low RBC indices in urinary schistosomiasis. Consequently, anaemia due to iron deficiency is a future concern for these children. Red cells haemolysis by the spleen and the corresponding urinary iron loss could have accounted for the reduced RBC indices among the cases [4]. It is also possible for S.* haematobium* in the bladder wall to reduce the uptake of iron during its pathogenesis through consumption of blood by the schistosomes or extrusion of schistosomal ova which causes urethra and bladder irritation [18].

Leukocytosis and eosinophilia have been implicated in the intensity of* S. haematobium* infection [4]. Consistent with previous studies, we observed a significant rise in absolute MXD and RDW levels (Table 1) among cases. A higher but insignificant total WBC and neutrophils have also been reported in this study. A significant rise in the percentage count of MXD is an indication of a corresponding rise in eosinophils, monocytes, and basophils. This may lead to a general immunological response as a number of systems employing antibodies were evoked to disturb the schistosomes [19].

Interestingly, a smaller percentage of children had heavy infections and were more anaemic than those with light infections (Table 2). However, significant reductions in the level of HGB in relation to degree of infection were not observed, which is consistent with the findings of Brito and others [20]. Slight increase in HGB level in heavily infected children is an indication that the community are getting aware of the clinical presentation of the infection and might have given their children some form of antihelminths, herbal preparations, or haematinics. Previous studies [21, 22] have shown that distribution of schistosomiasis now follows a negative binomial curve, with low worm burdens in most infected persons and only a few proportion harbouring heavy infections. This could have accounted for the low number of children with heavy infections.

Microcythaemia and hypochromia due to reduced MCV and MCHC in* Schistosoma* infected individuals advance the effect of schistosomiasis through its pathogenesis [18]. Multivariate logistic regression indicated that significantly low MCV and MCH, coupled with high MXD, were independently associated with* S. haematobium* infection among school aged children (Table 3). Our finding showed that school going children with* S. haematobium* infections were 5.57, 8.81, 2.07, and 2.50 times at risk of developing low MCV, MCH, and MCHC and high MXD, respectively. Previous work [6] affirms that low RBC indices are a common manifestation in* Schistosoma* infections. A significant rise in absolute eosinophil count is an independent risk factor for exacerbating the pathogenic effect of schistosomiasis [23].

The insignificance in the pattern of the infection albeit higher prevalence observed could be due to our small sample size. Clearly a follow-up study with a larger sample size prospectively would better assess the role of this infection on haematological profile. This study design would allow a better understanding of the profile over time and consequently give a true picture of the degree of exposure and the associated risk factors. Also, our findings may be limited by our inability to assess serum iron and ferritin levels among our study participants.

## 5. Conclusion

We conclude that* S. haematobium* infection creates an imbalance in the haematological profile. Among our study participants, we observed low HGB, HCT, MCV, MCH, and MCHC levels coupled with increased % MXD count and RDW-CV. We also report that low MCV, MCH, and MCHC as well as high % MXD count are independently associated with* S. haematobium* infection among our study participants. Treatment coupled with nutritional intervention, health education, and feeding programs should be considered to manage the deleterious effect of* S. haematobium* infections.

## Figures and Tables

**Table 1:** Haematological profile of study participants with *S. haematobium* infection.

Parameter	Cases	Control	*P* value
	(*N* = 50)	(*N* = 50)	
*Age (years)*	10.42 ± 2.16	11.18 ± 3.20	0.167
*Haematological profile*			
WBC × 10^3^/mm^3^	7.80 ± 0.34	7.26 ± 0.34	0.264
RBC × 10^6^/mm^3^	4.26 ± 0.08	4.36 ± 0.06	0.289
HGB (g/dl)	9.99 ± 0.13	11.36 ± 0.14	**<0.0001**
HCT (%)	32.04 ± 0.46	35.20 ± 0.42	**<0.0001**
MCV (*μ*m^3^)	75.87 ± 1.13	79.71 ± 1.61	**0.0053**
MCH (pg)	23.72 ± 0.43	26.21 ± 0.31	**<0.0001**
MCHC (g/dl)	31.25 ± 0.27	32.21 ± 0.19	** 0.005**
PLT × 10^3^/mm^3^	270.78 ± 13.93	242.56 ± 9.93	0.102
RDW-CV (%)	11.87 ± 0.21	11.21 ± 0.14	** 0.012**
*White cell differentials*			
LYM (%)	49.20 ± 1.32	50.05 ± 1.41	0.661
MXD (%)	13.49 ± 1.50	9.16 ± 1.01	** 0.018**
NEUT (%)	40.79 ± 1.64	37.31 ± 1.70	0.144

Values are presented as mean ± standard deviation; *N*: number of samples.

**Table 2:** Factors associated with *S. haematobium* infection stratified by heavy and light infections.

Variable	*S. haematobium* infection		*P* value
	Light	Heavy	
	(*N* = 37)	(*N* = 13)	
*Age (years)*	10.21 ± 2.08	10.50 ± 2.21	0.679
*Gender*			
Male	14 (37.8)	9 (69.2)	0.106
Female	22 (59.5)	5 (38.5)	
*Age range (years)*			
<10	12 (32.4)	5 (38.5)	0.974
10–12	18 (48.6)	7 (53.8)	
13–15	6 (16.2)	2 (15.4)	
*Water contact activity*			
Bathing	1 (5.4)	0 (0.0)	0.430
Swimming	24 (64.9)	10 (76.9)	
Washing	3 (8.1)	3 (23.1)	
Drinking	3 (8.1)	1 (7.7)	
Fishing	5 (13.5)	0 (0.0)	
*Urinalysis*			
Hematuria	0 (0.0)	13 (23.1)	**<0.0001**
Cloudy	12 (32.4)	0 (0.0)	
Clear	24 (64.8)	1 (7.7)	
*Haematological profile*			
WBC × 10^3^/mm^3^	7.48 ± 0.38	8.61 ± 0.72	0.138
RBC × 10^6^/mm^3^	4.25 ± 0.09	4.27 ± 0.13	0.902
HGB (g/dl)	10.24 ± 0.18	9.89 ± 0.17	0.224
HCT (%)	31.79 ± 0.59	32.69 ± 0.61	0.382
MCV (*μ*m)	75.3 ± 1.23	77.33 ± 2.54	0.425
MCH (pg)	23.54 ± 0.51	24.18 ± 0.82	0.516
MCHC (g/dl)	31.21 ± 0.34	31.38 ± 0.37	0.774
PLT × 10^3^/mm^3^	264.25 ± 17.08	287.57 ± 23.70	0.458
RDW-CV (%)	11.84 ± 0.25	11.95 ± 0.41	0.835
*White cell differentials*			
LYM (%)	50.12 ± 1.73	46.84 ± 1.51	0.269
MXD (%)	14.15 ± 1.83	11.78 ± 2.61	0.482
NEUT (%)	35.73 ± 2.08	41.38 ± 2.15	0.124

Values are presented as mean ± standard deviation and %; *N* = number of participants.

**Table 3:** Multiple logistic regression of factors associated with *S. haematobium* infections controlled for age and gender.

Factors	OR (95% CI)	*P* value
*Gender*		
Male	1 (reference)	
Female	1.15 (0.44–3.04)	0.779
*Age range*		
<10	1 (reference)	
10–12	1.71 (0.56–5.16)	0.344
13–15	2.47 (0.57–10.61)	0.226
16–18	0.00	—
*Water contact activity*		
Bathing	0.09 (0.00–2.03)	0.130
Swimming	0.21 (0.03–1.40)	0.107
Washing	0.99 (0.09–10.95)	0.991
Drinking	2.49 (0.09–71.21)	0.594
Fishing	1 (reference)	
*Haematological profile*		
HGB		
<11	3.75 (3.75)	0.111
>11	1 (reference)	
MCV		
<85	5.75 (1.19–27.81)	**0.030**
85–95	1 (reference)	
>95	6.89 (6.89)	0.109
MCH		
<27	8.81 (2.40–32.41)	**0.001**
27–33	1 (reference)	
MCHC		
<32	2.07 (0.93–4.60)	0.073
32–36	1 (reference)	
MXD		
<3	1.56 (0.53–4.59)	0.417
3–13	1 (reference)	
>13	2.50 (1.02–6.15)	**0.046**
RDW-CV		
<11	0.42 (0.18–0.99)	0.064
11–16	1 (reference)	
>16	1.01 (1.01)	0.178
